# Workplace Supported Recovery from Substance Use Disorders: Defining the Construct, Developing a Model, and Proposing an Agenda for Future Research

**DOI:** 10.1007/s41542-022-00123-x

**Published:** 2022-12-05

**Authors:** Michael R. Frone, L. Casey Chosewood, Jamie C. Osborne, John J. Howard

**Affiliations:** 1 Department of Psychology, University at Buffalo, The State University of New York, Buffalo, NY, United States; 2 Office of the Director, Office for Total Worker Health®, Centers for Disease Control and Prevention, National Institute for Occupational Safety and Health, Atlanta, GA, United States; 3 Office of the Director, Office for Policy, Planning and Evaluation, Centers for Disease Control and Prevention, National Institute for Occupational Safety and Health, Atlanta, GA, United States; 4 Office of the Director, Centers for Disease Control and Prevention, National Institute for Occupational Safety and Health, Washington, DC, United States

**Keywords:** Workplace, Workforce, Recovery, Addiction, Substance Use Disorder

## Abstract

Substance use disorders (SUDs) represent a critical public and occupational health issue. Therefore, understanding the process of SUD recovery has become an issue of growing importance among substance use and recovery professionals. Nonetheless, despite the acknowledged importance of employment for SUD recovery, little conceptual or empirical work exists on how the workplace might support or undermine SUD recovery. In this article, we address this limitation in several ways. First, to promote a better understanding of SUD recovery for occupational health researchers, we provide a brief overview of the nature of a SUD, prior definitions of SUD recovery, and general themes associated with the recovery process. Second, we develop a working definition of *workplace supported recovery*. Third, we present a heuristic conceptual model showing how the workplace might impact the SUD recovery process. Fourth, using this model and research from the substance use and occupational health literatures, we develop a series of general research propositions. These propositions highlight broad directions requiring more detailed conceptualization and empirical research to understand better how work conditions may support or undermine the process of employee SUD recovery. Our overarching goal is to motivate innovative conceptualization and research on workplace supported recovery from SUDs. Such research may inform the development and evaluation of workplace interventions and policies supporting SUD recovery and highlight the benefits of workplace supported SUD recovery for employees, employers, and communities. Research on this issue may allow occupational health researchers to impact a significant societal and occupational health issue.

Substance misuse and substance use disorders (SUDs) represent one of the critical public and occupational health issues of our time and impose enormous costs on American society (Office of the Surgeon General, 2016). In terms of annual economic costs, alcohol misuse and disorders cost approximately $249 billion in 2010 (Sacks et al., 2015). Additionally, illicit drug use and disorders, which involve illegal drug and nonmedical prescription medication use, cost approximately $193 billion in 2007 (National Drug Intelligence Center, 2011). Recently, Davenport et al., (2019) estimated that the direct and indirect economic cost of nonmedical opioid use alone amounted to $188 billion in 2019. These societal costs involve reduced employability, employment- and nonemployment-related lost productivity, health care expenses, morbidity and mortality, and criminal justice. Regarding mortality, the total number of U.S. drug overdose deaths rose from 16,849 to 1999 to 70,630 in 2019, primarily driven by increases in overdoses involving opioids, including dangerous synthetic opioids like fentanyl (Hedegaard et al., 2020). The average annual number of deaths from alcohol-associated causes from 2011 to 2015 was approximately 95,000 (Esser et al., 2020). Finally, Table 1 shows that 70.4% (about 13.6 million workers) of all adults with an alcohol or illicit drug use disorder are employed. Among employed adults, 8.7% (about 13.6 million workers) have current alcohol or illicit drug use disorders, 1.2% (about 1.9 million workers) receive some type of treatment annually for a SUD, and 8.5% (about 13.3 million workers) report that they are in recovery or have recovered from a past or present substance use problem. These details show that SUDs affect a relatively small proportion yet a significant number of individuals in the workforce (see Table 1).

Growing evidence supports that recovery from a SUD is achievable and benefits workplaces and society (Akhtar, 2019; McQuaid et al., 2017; Whitney, 2016). It is also essential to consider that attempts to recover from SUDs occur in environmental contexts that may support or undermine its initiation and sustainability. One environmental context that has a broad impact on people’s lives is employment. Although reviews of prior research provide evidence that various workplace factors are associated with substance misuse and SUDs (Ames & Moore, 2016; Frone, 2013, 2019), systematic research on the role workplaces may play in promoting or undermining the SUD recovery process is limited.

Given the importance of facilitating sustainable recovery among individuals with SUDs and the broad significance of employment in people’s lives, this article’s general goal is to present an initial overview of SUD recovery and how the workplace may broadly affect, either positively or negatively, employee recovery from SUDs. *The complex nature of SUDs and recovery may not be understood fully by employees, unions, employers, and many occupational health researchers. A better understanding of SUDs, SUD recovery, and the workplace’s potential influence can benefit the development of future research, effective policies and programs, and interventions to promote and sustain recovery among employees facing a SUD.*

To address this general goal, we begin by reviewing the nature of a SUD, definitions of SUD recovery, and summarize broad themes in the recovery process. Second, we offer a working definition of *workplace supported recovery*. Third, we present a conceptual model showing how the workplace might impact the SUD recovery process. Fourth, using this model and research from the addictions and occupational health literatures, we outline a series of general research propositions to guide future research on how work conditions may support or undermine the process of employee SUD recovery. Although some researchers have advocated for organizations to support workplace SUD prevention and recovery efforts as a form of corporate social responsibility (Radacsi & Hardi, 2014), the proposed research agenda may produce a robust evidence base to inform and support the development and evaluation of workplace policies and interventions that assist individuals with SUDs in initiating and sustaining their recovery efforts.

## What is a Substance Use Disorder?

In the fifth edition of the Diagnostic and Statistical Manual of Mental Disorders (DSM-5), the American Psychiatric Association presents 11 criteria for the diagnosis of a SUD that fall into four general categories (American Psychiatric Association, 2013): *physical symptoms* (tolerance—need to consume more to get the same effect and decreased effect with the same amount; withdrawal—experiencing withdrawal when use is reduced and using to avoid withdrawal; craving—strong desire to use a substance), *loss of control* (using more of a substance or for a longer period than intended; inability to cut down or stop using a substance), *forsaking meaningful activities* (spending much time using, obtaining, or being affected by a substance; giving up or reducing central life activities), and *problems related to use* (failure to meet role obligations; continued use despite social problems; continued use despite physical or psychological problems; using in physically hazardous situations). These criteria apply to all psychoactive substances (e.g., alcohol, cannabis, opioids, stimulants, and other drugs), representing chemical compounds that act primarily on the central nervous system, resulting in changes in consciousness, perception, emotion, cognition, or behavior (Frone, 2013, 2019).

Based on the DSM-5, a SUD can range in severity from mild (2–3 criteria) to moderate (4–5 criteria) to severe (6 or more criteria). The term addiction often is used to characterize the severe end of the SUD spectrum (National Institute on Drug Abuse, 2018; Office of the Surgeon General, 2016). The National Institute on Drug Abuse defines addiction as follows:

A chronic, relapsing disorder characterized by compulsive drug seeking and use despite adverse consequences. It is considered a brain disorder because it involves functional changes to brain circuits involved in reward, stress, and self-control, and those changes may last a long time after a person has stopped taking drugs (National Institute on Drug Abuse, 2018, p. 4).

Among individuals with a substance use disorder, national research in the U.S. on alcohol and cannabis use shows that approximately 53% have a mild disorder, 23% have a moderate disorder, and 24% have a severe disorder (Grant et al., 2015; Hasin et al., 2016).

## What is Recovery from a Substance Use Disorder?

The concept of *recovery* has gained momentum in the U.S. and other countries as a conceptual lens for understanding SUDs and their management (Kelly et al., 2018; Kelly & Hoeppner, 2015; National Academies of Sciences-Engineering-Medicine, 2016b; White, 2007b). In general terms, recovery refers to an “organizing framework for approaching substance use disorders as a chronic disorder from which individuals can recover, so long as they have access to evidence-based treatments and responsive long-term supports” (Office of the Surgeon General, 2016, p. 5 − 1). Recovery does not necessarily represent a cure. Instead, it represents a state of “being and becoming” (Sheedy & Whitter, 2009, p. 11), where some people may always consider themselves in recovery and others may eventually view themselves as fully recovered (UK Drug Policy Commission, 2008).

In more specific terms, the concept of “recovery”—being “in recovery” or being “recovered”—has been defined in many different ways (Ashford et al., 2019; el-Guebaly, 2012; Kelly & Hoeppner, 2015; White, 2007a). Table 2 provides several definitions of recovery, advanced by various organizations and researchers. Despite variation across these definitions, we observe among them and in related literature several general themes in the recovery process.

### Recovery Theme 1: Different Definitions of Recovery

For many researchers and recovery advocates, recovery begins with the development of long-term abstinence (i.e., sobriety) from all psychoactive substances, whereas others disagree with this general stance. Specifically, there exist two related points of discussion. The first point of discussion is whether long-term abstinence from all psychoactive substances is necessary for recovery. In other words, can individuals be in recovery if they maintain a level of use that is perceived to be less harmful or substitute a substance that they perceive to be less harmful? As shown in Table 2, the first two definitions suggest a need to achieve long-term abstinence from all substances (alcohol, illegal drugs, and nonmedical prescription drug use). The third definition refers to “voluntarily-sustained control over substance use.” The remaining two definitions do not mention abstinence or any level of use to broadly include who is in recovery or recovered (Recovery Science Research Collaborative, Ashford et al., 2019). Another reason is that a growing literature shows that the resolution of SUD symptoms is possible for many individuals who develop a level of use that they perceive to be less harmful, at least among those with mild to moderate SUDs (Stea et al., 2015; White, 2007a; Witkiewitz, 2020). A U.S. national survey found that some individuals with SUDs affirm this broader definition, reporting that 23.6% of those in recovery were not abstinent from their problematic substance(s), and 40.9% were not abstinent from all drugs and alcohol (Kelly, Abry, et al., 2018).

Although some definitions of SUD recovery propose that abstinence is required for a person to be in recovery, others either directly or indirectly suggest that both abstinence and nonabstinence goals are consistent with SUD recovery. Several overlapping terms characterize nonabstinence outcome goals (Rosenberg, Grant, & Davis, 2020). No matter the specific term used, they have three common elements: (a) limiting the amount consumed of the target substance, the frequency of consumption, and/ or the speed of consumption; (b) experiencing fewer if any harmful consequences of consumption; and (c) developing a sense of consumption-related self-efficacy (i.e., realistic confidence in using self-control behaviors to moderate use) (Rosenberg et al., 2020). Although abstinence is the most widely accepted and most beneficial goal for many individuals with SUDs, nonabstinence goals may be helpful when considered part of a broader harm-reduction strategy to motivate recovery efforts. Nonabstinence outcomes are beginning to be incorporated into randomized clinical trials of behavioral and pharmaceutical treatments and in the treatment guidelines of various countries and organizations for alcohol use disorders (e.g., World Health Organization) (Mann, Aubin, & Witkiewitz, 2017). However, these approaches may be safer and more practical when referring to alcohol use. The unpredictability of the illicit drug market due to adulteration and increasing potency (and therefore potential lethality) makes this approach challenging. Illicit drugs increasingly contain powerful synthetic opioids, such as fentanyl, or other harmful substances, alone or in uncharacterized mixtures. Synthetic opioids such as fentanyl were involved in an estimated 62% of drug overdoses in the 12 months ending in December 2020, based on provisional data (Ahmad et al., 2021). Given inherent and unpredictable risks in these scenarios, additional research into the utility of nonabstinence outcomes for substances other than alcohol is critical.

The second point of discussion relates to using medication-assisted therapy (MAT) during recovery. MAT represents the combination of approved medication (e.g., disulfiram or acamprosate for alcohol use disorders, methadone or buprenorphine for opioid use disorders, and naltrexone for both alcohol and opioid disorders) and psychosocial treatment, including peer support group participation (National Institute for Occupational Safety and Health, 2019; Office of the Surgeon General, 2016). Research shows that MAT may improve treatment outcomes by reducing cravings for and the euphoria experienced from a misused psychoactive substance (Connery, 2015; National Institute for Occupational Safety and Health, 2019; Office of the Surgeon General, 2016). However, despite MAT’s effectiveness, the recovery literature points out that individuals using MAT may experience stigma from some therapists and peer support groups (e.g., Alcoholics Anonymous, Narcotics Anonymous) because they are not considered to be abstinent and in recovery (Krawczyk et al., 2018; Office of the Surgeon General, 2016; Robinson & Adinoff, 2018; White, 2007a, 2011). These circumstances have resulted in peer support groups that allow full participation for individuals using MAT, such as Methadone Anonymous and Medication-Assisted Recovery Anonymous.

### Recovery Theme 2: Recovery is a Lived, Self-Directed, and Empowering Process that Involves Building Healthy, Productive, and Meaningful Lives

The recovery literature suggests that SUD recovery represents more than a complete or progressive move away from substance use and reduced associated harms. Importantly, it involves the accrual of positive benefits obtained by developing or rebuilding a healthy, productive, and meaningful life (Ashford et al., 2019; Betty Ford Institute Consensus Panel, 2007; Office of the Surgeon General, 2016; UK Drug Policy Commission, 2008; White, 2007a). Moreover, studies have found that recovery is sustainable and empowering and needs to be voluntary and self-directed, often with assistance (Sheedy & Whitter, 2009). Individuals in recovery acknowledge the importance of recognizing the need for change and being personally involved in and responsible for their recovery (Sheedy & Whitter, 2009).

### Recovery Theme 3: Recovery Represents an Individualized Growth Process

Recovery represents an individualized growth process because individual differences exist in the causes, severity, and outcomes of a SUD, internal and external resources available to address the disorder, and personal priorities (Ashford et al., 2019). Therefore, multiple pathways to recovery exist—religious, spiritual, and secular (UK Drug Policy Commission, 2008; White, 2007b). The recovery process can involve various forms of external assistance—formal treatment with or without medication assistance, formal peer support networks (e.g., alcoholics anonymous; narcotics anonymous; religious and culturally-based support groups, peer recovery coaching), or recovery support services (e.g., long-term care management, employment assistance, and housing support)—or it may involve no external assistance (i.e., unassisted or natural recovery). Kelly et al., (2017) found that among a U.S. national sample of individuals who resolved an alcohol or drug problem, 53.9% reported some form of assisted recovery, and 46.1% reported an unassisted or natural recovery.

The processes underlying SUDs and recovery may differ across various *special emphasis populations* (SEPs), representing “groups experiencing health disparities resulting in elevated risk to health, safety, and well-being” (Wagner & Baldwin, 2020, p. 1). SEPs include emerging adults, older adults, women, sexual/gender minorities, racial/ethnic minorities, and individuals with co-occurring disorders. Differences in the recovery process across SEPs can arise because of heterogeneity in physiological processes, culture, and exposure to social inequalities and disadvantages that may affect access to and the structure, acceptability, and success of various formal and informal treatments for SUDs (e.g., Wagner & Baldwin 2020).

Finally, the stage of recovery will differ across individuals based on the length of recovery and enhanced global functioning: (1) early recovery—1 month to 11 months, (2) sustained recovery—1 to 5 years, and (3) stable recovery—5 or more years (Betty Ford Institute Consensus Panel, 2007; el-Guebaly, 2012; O’Sullivan et al., 2019; White, 2007a). As the length of recovery increases, the annual risk of SUD recurrence drops substantially. Among those in stable recovery, the annual risk of recurrence approaches the annual risk of developing a SUD in the general population (Kelly et al., 2018).

### Recovery Theme 4: Recovery is Supported by a Chronic Care Model

SUD recovery benefits from embracing a chronic care model (Arria & McLellan, 2012; Sheedy & Whitter, 2009). The traditional acute care model promotes an expectation that single or short-term episodes of treatment will cure individuals with a SUD, even though such an expectation does not exist for other chronic health problems such as diabetes mellitus, hypertension, and asthma (McLellan et al., 2000; O’Brien & McLellan, 1996). However, treatment is considered ineffective when the recurrence of symptoms occurs for many individuals in the acute care model. In contrast, a chronic care model acknowledges that there is no cure for SUDs and allows for the possibility of multiple cycles of treatment, symptom remission, symptom recurrence, and reestablishment of treatment and self-management before a person achieves stable recovery (Arria & McLellan, 2012; Dennis & Scott, 2007; White & Kelly, 2001). Although brief interventions as part of acute care may benefit individuals with mild to moderate SUDs, those with severe SUDs (i.e., addiction) will benefit most from longer-term recovery-oriented systems of care (Office of the Surgeon General, 2016).

A cycle of treatment, symptom remission, symptom recurrence resulting from nonadherence with treatment regimens, and reestablishment of treatment and self-management is not unique to SUDs (Arria & McLellan, 2012). This cycle also is observed with other chronic medical disorders for which there is no cure. For example, the symptom recurrence rate due to nonadherence with treatment regimens for SUDs (40–60%) is similar to that for Type 1 diabetes (30–50%) and both hypertension and asthma (50–70%) (McLellan et al., 2000; O’Brien & McLellan, 1996). Therefore, contrary to beliefs sometimes held by the general public and some medical professionals, the recurrence of symptoms during or after SUD treatment is not a sign of failure (Dennis & Scott, 2007). Instead, it is consistent with other chronic disorders requiring a lifelong commitment to treatment regimens and indicates that treatment needs to be reinstated or adjusted or alternate treatment may be needed (Arria & McLellan, 2012; Office of the Surgeon General, 2016).

### Recovery Theme 5: Recovery is Supported by Reducing Stigma and Promoting Accurate and Positive Portrayals of Persons with SUDs

Having a SUD or being in recovery from a SUD can lead to broad exposure to stigma and discrimination by the general public and healthcare providers, and may be experienced in many environmental contexts, including the workplace (Livingston et al., 2012; Rasinski et al., 2005; Roche et al., 2019; Schomerus et al., 2011; van Boekel et al., 2013; Vilsaint et al., 2020; Yang et al., 2017). This stigma and discrimination may undermine access to treatment, treatment initiation, and sustainability of recovery efforts. To avoid perpetuating stigma and discrimination and allow individuals with active SUDs or in recovery to transcend stigma, White & Kelly (2001) suggested that discussions of SUDs as a chronic condition should provide the following disclaimers:

“Characterizing addiction as a ‘chronic disorder’ does *not* mean that (1) all AOD [alcohol and other drug] problems have a prolonged, progressive course, (2) all persons with AOD problems need specialized professional treatment and long-term posttreatment monitoring and support, (3) all persons suffering from substance dependence will relapse repeatedly and require multiple treatment episodes, (4) there is minimal hope for full, long-term recovery, or (5) that persons with a chronic form of substance dependence have any less personal responsibility for illness self-management than those with diabetes or hypertensive disease” (p. 69).

Several articles from a national study of U.S. adults in recovery support the broad experience of discrimination and many of these disclaimers. Vilsaint et al., (2020) reported a high prevalence of perceived discrimination among individuals in recovery (discussed in more detail later). Kelly et al., (2017) found that 46.1% of those who resolved a substance use problem reported an unassisted recovery. Kelly et al., (2017) also reported that 64.5% of participants reported that it had been five or more years (35.2% reporting 5–15 years and 29.3% reporting 15 + years) since they resolved their substance use problem, indicating that stable recovery is common. Kelly et al., (2019) reported that the median number of recovery attempts to successful problem resolution was “surprisingly low and may offer hope to those struggling with AOD problems” (p. 1544). Specifically, this study found that the median number of serious attempts before resolving a substance use problem was two attempts, with 50% of individuals falling in a range of one to four attempts, and 13% of individuals reported problem resolution without a serious effort. In addition, the median number of attempts before successful problem resolution did not differ substantially across alcohol, cannabis, opioids, stimulants, and other drugs. Nonetheless, for individuals with severe SUDs, more recovery attempts were reported before the successful resolution of a substance use problem, indicating the benefits of a chronic care recovery model.

### Recovery Theme 6: Recovery is Supported and Sustained by the Development of Recovery Capital

Recovery capital is critical to an individual’s ability to recover from a SUD and represents the totality of internal and external resources that a person can draw upon to initiate and sustain the recovery process (Cloud & Granfield, 2008). Individual differences exist in the level of recovery capital when initiating recovery efforts and the rate of developing recovery capital during the recovery process. Researchers have identified various recovery capital dimensions, though considerable overlap exists across taxonomies (Hennessy, 2017). Cloud and colleagues (Cloud & Granfield, 2008; White & Cloud, 2008) proposed the most widely used taxonomy of recovery capital, shown in Table 3.

Conceptual and empirical research on recovery capital has focused primarily on the benefits bestowed by various types of recovery capital. However, Cloud & Granfield (2008) noted that although the general classes of recovery capital can support SUD recovery, they also can undermine recovery efforts under various circumstances, which they referred to as negative recovery capital. For example, developing strong relationships with non-substance-using peers (i.e., positive recovery capital) can help individuals become and remain abstinent. In contrast, maintaining strong relationships with peers who use substances (i.e., negative recovery capital), though providing positive outcomes such as a sense of belonging and identity, can create a hazard that undermines the initiation and sustainability of recovery efforts. Therefore, successful recovery efforts may depend on the net effect of simultaneously reducing negative recovery capital and increasing positive recovery capital.

## What is Workplace Supported Recovery?

As the above discussion suggests, recovery from a SUD can be a complex, dynamic, and heterogeneous process. However, one recurring theme in the recovery literature is the importance of employment (e.g., Borrelli et al., 2017; Brown & Ashford, 2019; Grover & Paylor, 2010; Laudet & White, 2010; Monaghan & Wincup, 2013; Room, 1998; Worley, 2017). In essence, employment represents a critical environmental context in which employees’ SUD recovery process unfolds. Recognizing the significant role employment and employers can play in individuals’ recovery efforts, there are efforts at the national and state level and among recovery organizations to promote workplace supported recovery (https://www.cdc.gov/niosh/topics/opioids/wsrp/resources.html). However, few attempts have been made to define workplace supported recovery.

These efforts to promote workplace supported recovery have primarily focused on the resources provided by employment that can support recovery efforts. This focus is understandable because employment can provide critical manifest (e.g., income, paid time off, health benefits, and retirement benefits) and latent (e.g., time structure and regular activity, social contacts, purpose and meaning, development of skills, goal achievement, and status and identity) resources (Jahoda, 1981, 1982). Nonetheless, it is also essential to consider that employment exposes employees to workplace hazards associated with substance misuse and SUDs, such as work stressors, substance availability, and prescriptive workplace substance use norms (Ames & Moore, 2016; Frone, 2013, 2019). In addition to contributing to or perpetuating SUDs, these workplace hazards may also undermine the initiation and sustainability of SUD recovery efforts. Therefore, any definition or discussion of workplace supported recovery may benefit from considering both the employment-related hazards and supports that can affect SUD recovery.

Given the minimal research exploring the components of workplace supported recovery, we propose the following working definition of workplace supported recovery from SUDs:

An integrated set of evidence-based interventions and policies that (a) reduce workplace hazards promoting the development or perpetuation of substance use disorders and undermining recovery from substance use disorders; (b) increase workplace supports preventing the development or perpetuation of substance use disorders and facilitating recovery from substance use disorders; (c) help employees maintain or regain employment during recovery; and (d) promote overall growth and well-being among employees, work organizations, families, and communities.

This definition highlights several broad assumptions underlying workplace supported recovery. The first assumption is that workplace efforts to address SUDs and recovery require efforts on several fronts. Workplaces may consider reducing hazards and providing support in an integrated effort to address SUDs, support unassisted and assisted treatment and recovery efforts, and promote overall well-being among employees. This approach is consistent with a critical recovery theme that SUD recovery reflects more than sustained abstinence resulting from workplace hazard reduction. It also involves the accrual of positive benefits obtained by developing or rebuilding a healthy, productive, and meaningful life resulting from workplace support and health promotion. The reduction of workplace hazards and strengthening of workplace supports promoting recovery efforts and well-being are aligned with the National Institute for Occupational Safety and Health (NIOSH) *Total Worker Health®* (TWH) approach. This approach calls for policies and practices that integrate the reduction of workplace hazards with the promotion of workplace supports to improve workers’ overall well-being (Schill et al., 2019).

The second assumption underlying this definition is that the benefits of workplace supported recovery efforts accrue to more than the affected employees. Coworkers, employers, families, and the communities where employees with SUDs live also may benefit. Moreover, for workplaces and organizations, reducing workplace hazards and promoting workplace supports affecting well-being may produce workplace benefits beyond reducing SUDs. These actions may affect all workers in ways that improve job satisfaction, employee attendance and engagement, job performance, worker safety, organizational commitment, and employee retention.

A final assumption to this definition is also critical. Workplace supported recovery programs and approaches may not look the same in every organization or every occupation or industry. Supporting workers in treatment and/or in recovery from a SUD may require customized strategies, especially for those performing hazardous or safety-sensitive work. Qualified occupational healthcare providers can help to evaluate job roles with appropriate consideration given to any health challenges faced by the worker occupying the role. They can then make case-by-case determinations about specific and necessary restrictions or job limits. Reasonable accommodations should be considered and implemented by the employer when possible. Additionally, given the lack of recovery research on nonabstinence approaches, especially for SUDs involving illicit drug use, workplace supported recovery is generally characterized as abstinence for this paper. This may be especially critical when applied to workers performing hazardous work or in those who have safety-sensitive job duties and responsibilities.

## Is Substance Use Disorder Recovery Associated with Positive Employment Outcomes?

As noted earlier and shown in Table 1, most adults in the U.S. with alcohol or illicit drug use disorders are employed, and a relatively small proportion, yet substantial number of individuals in the workforce report a current SUD or that they are in recovery or have recovered from a substance use problem. So, do individuals in treatment and recovery report better work-related outcomes? Although little research has explored this question, there is evidence that SUD treatment and recovery may be associated with positive work outcomes.

For example, Slaymaker & Owen (2006) found that among employed individuals admitted to residential treatment, 65% reported continuous abstinence at six months, 51% were continuously abstinent at 12 months posttreatment, and most individuals regularly attended Alcoholics Anonymous meetings. At 12 months posttreatment, 65% of individuals continued to work for the same employer. In addition, the percentage of individuals reporting unplanned absences decreased from 78% before treatment to 30% at the 12-month follow-up. Further, substantial reductions from pretreatment to 12-month follow-up were reported in the number of problem days at work in the past month (5.20 days vs. 0.14 days), and the proportion of individuals disciplined at work (22.2% vs. 6.5%), reporting their job was in jeopardy (18.2% vs. 5.2%), and losing their job during the preceding 12 months (men only, 24% vs. 7%). Similarly, among employed individuals admitted to inpatient treatment, Arbour et al., (2014) found that there were substantial reductions from pretreatment to 6-month follow-up in the number of days absent among people who use substances (alcohol: 7.6 days vs. 0.8 days; drugs: 16.6 days vs. 5.1 days), days tardy (alcohol: 8.5 days vs. 0.9 days; drugs: 17.3 days vs. 1.61 days), and days unproductive (alcohol: 20.9 days vs. 4.2 days; drugs: 43.0 days vs. 12.1 days).

Using data from the 2012 to 2014 National Survey of Drug Use and Health, Goplerud et al., (2017) reported that workers in recovery reported better annual attendance and work stability (absent: 9.5 days; more than one employer: 23%) than those with a current SUD (absent: 14.8 days; more than one employer: 36%) and similar outcomes to those in the general workforce who never had a SUD (absent: 10.5 days; more than one employer: 25%). In addition, workers in recovery reported a lower prevalence of past-year affective disorders (serious psychological distress: 3%; anxiety disorder: 6%; depression: 7%) than those with a current SUD (serious psychological distress: 12%; anxiety disorder: 11%; depression: 11%) and similar outcomes to those in the general workforce without a SUD (serious psychological distress: 4%; anxiety disorder: 5%; depression: 6%). Although healthcare utilization was roughly equivalent across the three groups of workers, healthcare costs per employee per year were lower among workers in recovery ($1,638) than those with a current SUD ($2,197) and similar to those in the general workforce without a SUD ($1,729).

Finally, four Life in Recovery Surveys conducted in the U.S. (Laudet, 2013), Canada (McQuaid et al., 2017), UK (Best et al., 2015), and Australia (Best, 2015) assessed reported changes from active addiction to recovery on several outcomes spanning multiple life domains (e.g., family/social life, finances, health, legal, and employment). Each survey used a broad national convenience sample of adults reporting that they were (a) in recovery, (b) recovered, (c) used to have an alcohol or drug problem but no longer have a problem, or (d) were in medication-assisted recovery. In all four studies and across all life domains, the percentage of individuals reporting positive experiences increased, and the percentage reporting negative experiences decreased during recovery compared with active addiction. Table 4 shows the prevalence of positive and negative employment-related experiences during active addiction and recovery. Also, across all employment and non-employment outcomes, the increases in positive experiences and decreases in negative experiences became larger as recovery length increased.

Although the research exploring the association between SUD recovery and work outcomes is sparse, these studies provide initial evidence that recovery may be associated with substantial productivity improvements and lower healthcare costs that benefit both employees and employers. However, future research on this issue must use stronger study designs to provide more robust causal evidence for employers, including longitudinal observational studies and randomized clinical trials.

## The Impact of the Workplace on Substance Use Disorder Recovery

Despite the societal and employer consequences arising from SUDs and efforts to promote SUD recovery in the workforce, no systematic research exists on how the work environment might impact the process of SUD recovery. Therefore, in this section of the article, we present and describe a heuristic conceptual model showing how the workplace might impact the SUD recovery process. We then use this model and relevant research from the substance use and occupational health literatures to develop a series of general research propositions. These propositions highlight broad directions requiring more detailed conceptualization and empirical research to understand better how the work environment may impact SUD recovery efforts.

## Conceptual Model of Workplace Supported Recovery

**Fig. 1:**
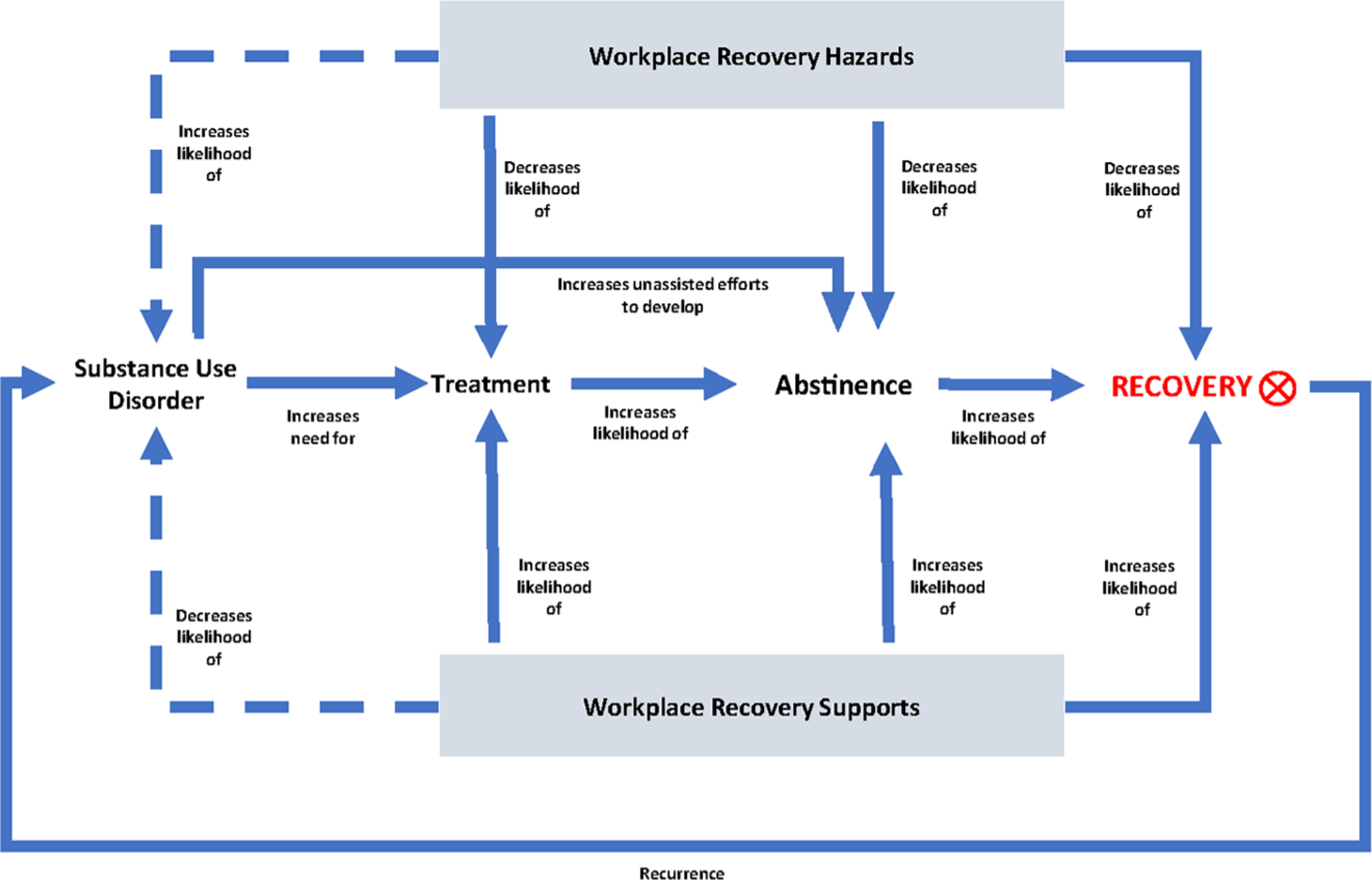
Model of Workplace Supported Recovery for Substance Use Disorders

At the center of the model in Fig. 1 is a general depiction of the recovery process. The first indirect path from a SUD to recovery through treatment represents assisted recovery. Specifically, having a SUD motivates entry into treatment, representing any combination of formal treatment with or without medication assistance and formal peer support networks (e.g., alcoholics anonymous, narcotics anonymous). Treatment then increases the likelihood of abstinence, which increases the possibility of reduced clinical symptoms and broader recovery efforts and outcomes. The second indirect path from a SUD to recovery represents unassisted or natural recovery. In this path, having a SUD leads to unassisted abstinence, which increases the likelihood of reduced clinical symptoms and broader recovery efforts and outcomes. Finally, there is a feedback path indicating that if recovery efforts are interrupted, a recurrence of the SUD may occur, thereby beginning a new cycle.

The model also shows where work conditions might impact the SUD recovery process. Although the work environment may play a role in the development or perpetuation of substance misuse and SUDs, depicted by the two broken lines in Fig. 1, we do not focus on those associations. The reader can consult recent reviews of the employee substance use literature for more information (Ames & Moore, 2016; Frone, 2013, 2019). Nonetheless, this research can inform us about potential workplace effects on the recovery process after a SUD develops and is discussed later in this context. The solid lines emanating from the workplace recovery hazards and supports boxes indicate that aspects of the work environment can decrease or increase the likelihood of (a) initiating and completing treatment and maintaining involvement in peer-support groups, (b) achieving and sustaining abstinence, and (c) maintaining involvement in long-term care, if required, rebuilding parts of one’s life damaged by a SUD, and developing and sustaining improvements in physical, mental, and social well-being that is the essence of recovery. Given the lack of systematic research on the association between the workplace and SUDs recovery, we do not know which workplace hazards and supports will have the greatest impact on the SUD recovery process. Below, we will explore some potential candidate workplace hazards and supports.

Finally, as evident in the discussion below, no overarching theory exists to explain how the workplace relates to the SUD recovery process. The associations of the various workplace hazards and supports to the dimensions of the SUD recovery process are couched in different theoretical perspectives. For example, the association of negative work conditions to dimensions of the SUD recovery process may be understood within the context of instrumentalization theory. In contrast, the association of workplace substance use norms to the SUD recovery process may be understood within the context of ritual theory. The various theories that help us to understand and design studies to test these individual associations are referred to as unit theories (Cronin et al., 2021). In contrast, a programmatic theory represents the integration of unit theories to understand the broader issue of employee SUD recovery (Cronin et al., 2021). Moreover, unit theories are the building blocks of programmatic theory, and the latter is the direct building block for application (i.e., workplace interventions) (Cronin et al., 2021). Although not a programmatic theory per se, the heuristic model presented in Fig. 1 and the discussion below can help researchers take a holistic view of the various unit associations that need to be understood to develop a broader theoretical understanding of the workplace supported recovery process.

## Agenda for Future Research on Workplace Supported Recovery

Although employment may affect SUD treatment and recovery, research on this issue would benefit from assessing the more granular work environment features instead of only broad employment features. A study by Sahker et al., (2019) illustrates why this is important. These researchers analyzed data from 8,925 individuals who participated in various publicly-funded SUD treatment modalities in Iowa. The researchers explored the associations of several broad employment variables assessed at pretreatment (employment status, employment tenure, and total income) to treatment completion at discharge (i.e., not dropping out of treatment) and alcohol use six months post-discharge. The authors predicted that these broad employment variables would represent employment-related recovery supports and, therefore, be related to treatment completion and lower levels of alcohol use. As expected, being employed full-time (versus being unemployed or out of the labor force), employment tenure, and higher income were associated with higher treatment completion rates. However, contrary to expectation, these same broad employment variables were associated with similar or higher levels of alcohol use at six months post-discharge rather than lower levels.

Full-time employment, longer employment tenure, and higher income may represent motivational resources for treatment completion to maintain employment. However, these variables do not tell us if a higher likelihood of treatment completion is an outcome of merely being employed or some more specific work environment features. Likewise, the findings involving post-discharge alcohol use may not have been unexpected had the researchers examined work environment hazards and (lack of) work-related support. In other words, the broad employment variables do not provide information on how specific dynamics in the work environment might affect post-discharge alcohol use. Instead, a more granular assessment of the pretreatment and posttreatment work environment is needed to understand the unexpected finding. This lack of information on the work environment’s granular dimensions also makes it challenging to identify specific recommendations for workplaces and SUD treatment and recovery support providers to leverage the workplace to ameliorate SUDs.

Therefore, we explore work conditions that may influence SUD treatment and recovery. These work conditions, derived from the literature on employee substance use, occupational health, and SUDs, provide an initial agenda for future workplace-supported recovery research. Before workplace interventions to promote and sustain SUD recovery can be developed and evaluated thoroughly, we need a better understanding of (a) the workplace hazards that need to be eliminated or mitigated and (b) the workplace supports that could be provided or strengthened to maximize workplace supported recovery. Next, we discuss these potential workplace recovery hazards and supports.

### Workplace Recovery Hazards

#### Negative work conditions.

Drug instrumentalization theory proposes that individuals use psychoactive substances to meet various motivational goals (Muller, 2020; Muller & Schumann, 2011). The evolutionary foundation of instrumentalization theory is that nonaddictive substance use in specific microenvironments can confer benefits by solving proximate adaptive problems (Muller, 2020; Muller & Schumann, 2011). One motivational goal of substance use is self-medication. The proximate adaptive problem solved by self-medication is the transition between different mental states—for example, reducing negative mental states associated with adverse work conditions and promoting more positive mental states (Muller, 2020). However, instrumentalization theory also proposes that some vulnerable individuals overuse substances for self-medication and other goals resulting in escalated use. This chronic increase in substance exposure (higher frequency and quantity of use; use of multiple drugs) among vulnerable individuals leads to changes in brain function that cause behavioral inflexibility and compulsive use, resulting in addiction (i.e., severe SUD) (Muller, 2020; Muller & Schumann, 2011).

Based on instrumentalization theory, the process underlying work-related self-medication begins with exposure to negative work conditions (e.g., excessive demands, bullying, job insecurity, hazardous physical work environment, work-related injuries), which leads to strain (e.g., job dissatisfaction, negative affect, negative perseverative cognition, fatigue, and pain), which then motivates psychoactive substance use to reduce the experienced strain (Frone, 2019). Research supports indirect associations between negative work conditions and increased substance use via various forms of strain, though there is growing evidence that these indirect associations are conditional. In other words, more robust and consistent associations exist between negative work conditions and hazardous substance use (heavy use, workday use) among vulnerable workers predisposed to self-medicate, such as those with low self-control, high neuroticism, and high positive outcome expectancies supportive of self-medication (Frone, 2013, 2019; Grandey et al., 2019).

Exposure to adverse work conditions and resulting strain may exacerbate substance use among those with a current SUD and lead to a recurrence of substance use among those in recovery (Becker, 2012; Sinha, 2012). In other words, individuals with an active SUD may be less likely to initiate treatment and abstinence, whereas those in recovery may fail to maintain these behaviors. The few studies that have examined these issues found that work-related stressors and negative affect reduced the likelihood of quitting and increased the likelihood of stress-triggered recurrence of substance use among those who had quit (Albertsen et al., 2006; Siahpush & Carlin, 2006; Strowig, 2000).

Another avenue by which negative work conditions can undermine SUD recovery is weakening recovery capital and the broader well-being outcomes individuals attempt to develop in recovery. As noted earlier, SUD recovery involves building or rebuilding the internal and external resources (recovery capital) that people can draw upon to support the recovery process and develop healthy, productive, and meaningful lives. Research shows that a broad range of negative work conditions can undermine the human, social, and physical capital beneficial for recovery (see Table 3), as well as the broader aspects of work-related (e.g., job satisfaction), physical, mental, and social well-being (Bannai & Tamakoshi, 2014; Barling et al., 2005; Chiaburu & Harrison, 2008; Clarke, 2019; Frone & Blais, 2020; Gisler et al., 2018; Goh et al., 2017; Jackson & Frame, 2018; Lundberg, 2015).

##### Research Proposition 1

Negative work conditions will decrease the likelihood of (a) initiating unassisted and treatment-assisted recovery efforts, (b) achieving or sustaining abstinence, and (c) achieving broad recovery outcomes such as growth in positive recovery capital and improved physical, psychological, and social well-being.

#### Workplace physical availability.

Availability theories of substance use (Single, 1988; Smart, 1980) propose that physical access to substances and ease of use may promote higher levels of substance use and misuse, which can increase the likelihood of SUDs and act as a trigger for craving and use among those with an active SUD or in recovery. Physical availability of alcohol and drugs at work represents the ease of obtaining these substances at work and bringing them into the workplace or using them during the workday (Ames & Moore, 2016; Frone, 2012, 2013, 2019). Research indicates that the physical availability of psychoactive substances at work, including access in specific jobs (e.g., bartenders, nurses, physicians, pharmacists), is associated with substance use and misuse during the workday and away from work (Ames & Moore, 2016; Frone, 2013, 2019). Although research on the association of work-place substance availability to other dimensions of the recovery process is lacking, general substance use research supports an association between physical availability of substances and both treatment dropout (Jacobson, 2004) and posttreatment recurrence of substance use during recovery (Tate et al., 2006; Tucker et al., 1991). Based on this limited research, we propose that among employees with an active SUD, workplace physical availability of substances may be used to rationalize and support their substance use. Also, among people with an active SUD or in recovery, work-place physical availability may act as an envirnmental trigger for substance use and undermine a person’s motivation to enter or remain in recovery, respectively, whether treatment-assisted or unassisted. This process may further erode the recovery capital needed to initiate and sustain the SUD recovery process.

##### Research Proposition 2

Physical availability of substances at work will decrease the likelihood of (a) initiating unassisted and treatment-assisted recovery efforts, (b) achieving or sustaining abstinence, and (c) achieving broad recovery outcomes such as growth in positive recovery capital and improved physical, psychological, and social well-being.

#### Workplace substance use norms and rituals.

General theories of social influence and learning (Cialdini & Trost, 1998) suggest that perceived social norms may be critical antecedents of substance misuse and disorders. Research shows that social norms supporting alcohol and illicit drug use before or during the workday predict higher alcohol and illicit drug use on and off the job (Ames & Moore, 2016; Frone, 2013, 2019). For example, a study of newly hired sales professionals in China showed that exposure to heavy drinking among veteran sales professionals and clients was associated with heavy drinking among the new sales professionals during client interactions, which was subsequently associated with heavy drinking in their personal lives (Liu et al., 2015).

Among individuals with an active SUD, exposure to workplace social norms supporting substance use may trigger craving and substance use. Workplace norms may create an aura of acceptability and normality supporting an individual’s substance use. Such workplace norms may undermine motivation to initiate unassisted or assisted attempts to quit or control one’s substance use. Among individuals in recovery from a SUD, exposure to workplace norms supporting substance use may trigger cravings that lead to a recurrence of substance use and the SUD.

Based on ritual theory (Collins, 2004), after-work alcohol use among coworkers can be viewed as a lifestyle ritual that signals the transition from work to leisure, marks the social boundary of inclusion or exclusion of coworkers in one’s after-work social group, and strengthens social identities and bonds among group members. Instrumentalization theory (Muller, 2020; Muller & Schumann, 2011) also suggests that after-work drinking rituals involve two substance-related motivational goals—self-medication to enable the transition from a stressful workday to leisure and the desire to improve social interactions.

A qualitative study by Buvik (2020) found that participants viewed after-work drinking with coworkers as a way of unwinding and disengaging from the demands and stresses of the workday and marking the transition to nonwork or leisure. Also, participants emphasized how after-work drinking could build camaraderie and cohesion among coworkers and served as a means of enhancing teambuilding. Although participating in after-work drinking was seen as having positive outcomes for coworkers and the work environment, Buvik (2020) noted that “a work environment with high alcohol consumption can be challenging for those who refrain from drinking alcohol” (p. 4). In Buvik’s (2020) study, participants reported that failing to participate in after-work drinking could be viewed as a rejection of coworkers resulting in exclusion from after-work and workplace social life. Such social exclusion could pressure individuals to join after-work drinking groups, thereby interfering with the initiation of recovery efforts among those with an active SUD and could trigger craving and the recurrence of drinking among those in recovery from a SUD. Social exclusion also may undermine the social recovery capital needed for broader recovery efforts and the emotional and social well-being that make up SUD recovery.

Although Buvik’s (2020) qualitative observations regarding ritualistic alcohol use have potential implications for employee SUD recovery, it is important to understand that the ritualistic use of alcohol on and off the job to socialize new workers, build solidarity and camaraderie among coworkers, sanction those who do not participate through social exclusion, and the potential of ritualistic drinking to increase the risk of alcohol use disorders and undermine recovery has been documented since the early 19th century. For an intriguing review of this issue and a qualitative study on the evolution of New York City tunnel worker’s (also known as sandhogs) intemperate drinking culture to a temperate or sober drinking culture, see Sonnenstuhl (1996).

Despite a lack of systematic research on the association of workplace substance use norms and rituals to the SUD recovery process, research in the addictions area shows that general social norms and social pressure can undermine recovery by increasing posttreatment recurrence of substance use (Marlatt & George, 1984; Ramo & Brown, 2008; Strowig, 2000; Tucker et al., 1991; Yong et al., 2018) and reducing the likelihood and frequency of attending peer support groups offering long-term recovery support (e.g., Alcoholics Anonymous, Narcotics Anonymous) (Davey-Rothwell et al., 2008).

##### Research Proposition 3

Workplace social norms and work-related rituals that support substance use will decrease the likelihood of (a) initiating unassisted and treatment-assisted recovery efforts, (b) achieving or sustaining abstinence, and (c) achieving broad recovery outcomes such as growth in positive recovery capital and improved physical, psychological, and social well-being.

#### Stigma.

Stigma represents a personal attribute or label (e.g., addict, alcoholic, drunk, druggie, stoner), conveying that a person possesses a fundamental and profoundly discrediting flaw (Goffman, 1963). This perception of a flaw leads to a set of negative attributions and stereotypes concerning those with a SUD, such as being perceived to be dangerous, unpredictable, worthless, poorly motivated, failing to meet primary role responsibilities, unemployable, solely responsible for one’s condition, unable to gain control of one’s life, and morally deficient (National Academies of Sciences-Engineering-Medicine, 2016a; Nieweglowski et al., 2018; Schomerus et al., 2011; van Boekel et al., 2013; Yang et al., 2017). These negative perceptions can lead to prejudice, discrimination, harassment, social exclusion, limited opportunities to participate fully in employment and other life roles, reduced public financial support for SUDs treatment, and increased support for legally coerced treatment (Hinshaw & Stier, 2008; Krupa et al., 2009; Roche et al., 2019; Schomerus et al., 2011; Stuart, 2004). Research also demonstrates that SUDs are highly stigmatized compared with other psychiatric disorders (major depression, schizophrenia) by both the general public, including employees and employers, and health professionals (Corrigan & Nieweglowski, 2018; Jorm & Oh, 2009; Lloyd, 2013; National Academies of Sciences-Engineering-Medicine, 2016a; Nieweglowski et al., 2018; Roche et al., 2019; Schomerus et al., 2011; van Boekel et al., 2013; Yang et al., 2017).

The stigmatization of SUDs can be rooted in an ingroup/outgroup dehumanization process, where those in the ingroup (individuals without SUDs) often believe that those in the outgroup who possess a stigma (e.g., SUD) lack distinctly human characteristics based on attributions and stereotypes described earlier (Leyens et al., 2000; Schroeder & Epley, 2020). These distinctly human characteristics include mental functioning (e.g., cognition, rationality, self-control), secondary emotions (e.g., compassion, happiness, shame, remorse, melancholia), and higher-order psychological needs (self-esteem, self-actualization) (Leyens et al., 2000; Schroeder & Epley, 2020).

A study by Schroeder & Epley (2020) illustrates the dehumanization of individuals with SUDs. They asked individuals to rate the importance of three levels of needs for several human targets and one nonhuman primate target. The targets were self, close friend, lawyer, elderly person, child, homeless person, drug addict, and chimpanzee. The three levels of needs were low-level physiological needs (eating, drinking to avoid thirst, sleeping), middle-level safety/belonging needs (feeling safe, predictability, getting affection from others, feeling like one belongs), and higher-order psychological needs (self-esteem and self-actualization—feeling respected, achieving personal and professional goals, living with meaning and purpose in life, realizing one’s potential in life). The results showed that high-level psychological needs were rated less important for drug addicts and chimpanzees than for the remaining six targets. In addition, the importance of low-level physiological needs and middle-level safety and belonging needs were rated lowest for drug addicts compared with all other targets, including chimpanzees. Schroeder & Epley (2020) speculated that all needs, including low-level physiological needs required for survival, were rated as less important for drug addicts because they are “presumed to find only one need important—likely satisfying their drug addiction—at the expense of all other psychological and physical needs” (p. 5).

Evidence suggests that such broadly dehumanizing perceptions of individuals with severe SUDs are not borne out. Like people without SUDs, most individuals with SUDs have aspirations for healthy, happy, secure, and productive lives (Brady, 2012; Thom, 2010). Indeed, such aspirations underlie the voluntary and self-directed nature of successful recovery efforts (see Recovery Theme 2). Schroeder and Epley’s (2020) findings are noteworthy because they underscore the level of stigmatization and misunderstanding directed at individuals with severe SUDs that increases the difficulty of overcoming their addiction and fulfilling their dreams and aspirations.

In the workplace, the stigma associated with an active SUD can profoundly impact employees, as evidenced by difficulty obtaining or maintaining employment, holding low quality and precarious employment, low income, constraints that undermine job performance and promotion opportunities, social exclusion, and an inability to obtain employer accommodations (Hakulinen et al., 2019; Krupa et al., 2009; National Academies of Sciences-Engineering-Medicine, 2016a; Nieweglowski et al., 2018; Roche et al., 2019; Stuart, 2004). Several studies provide evidence of SUD stigma in the workplace. The National Safety Council surveyed organizational decision-makers in U.S. organizations with 50 or more employees in 2017 and 2019 (National Safety Council, 2017, 2019). In the 2017 NSC survey, employers reported their general perceptions of prescription drug misuse. Consistent with contemporary views of SUDs, the majority of employers agreed with statements supportive of treatment and recovery and less stigma—misuse of prescription drugs represents “a sign of addiction” (80%) and “a disease that should be treated like any other chronic disease” (71%). Nonetheless, a substantial proportion of employers agreed with statements characterizing stigma that may undermine treatment and recovery—the misuse of prescription drugs represents “a justifiable reason to fire an employee” (65%), “a signal that an employee cannot be trusted” (43%), and a “moral/ethical failing” (42%).

In the 2019 NSC survey, employers reported the approach that best reflects how their organization would handle an employee misusing any of nine drugs. The nine drugs fall into three categories—alcohol, legally obtained prescription drugs (opioids, stimulants, benzodiazepines), and drugs illegal at the federal level (state-legal marijuana, illicit marijuana, illicitly obtained prescription opioids, heroin/fentanyl, other illicit drugs). Less than half of employers endorsed a non-stigmatizing response supportive of treatment and recovery—returning the employee to their original position after appropriate treatment—44% for alcohol, 40% for prescription drugs, and 26% for illegal drugs. Also, more than half of employers endorsed a response that may foster stigma and undermine the initiation of treatment and the sustainability of recovery—carefully monitor employee for the rest of his/her employment with the company, relocate the employee to a position of lesser responsibility, or dismiss the employee—52% for alcohol, 51% for prescription drugs, and 69% for illegal drugs.

Manning & White (1995) asked employers whether they believed the medical diagnosis in sick notes for employees on sick leave. Believing that an employee was legitimately off work sick because of physical illness was reported by 82% of employers. However, only 63% of employers believed that an employee was genuinely ill with a psychological illness (depression, schizophrenia, alcoholism). When asked if they would dismiss an employee who developed a disorder during their employment, the proportion saying no was 13% for alcoholism, 15% for schizophrenia, and 23% for depression. These results suggest that relatively few employers would retain an employee who developed a disorder, and consistent with information discussed earlier, those with a SUD are among the most stigmatized.

Furthermore, workplace stigma extends beyond those with active SUDs, including individuals in recovery. Although Baldwin et al., (2010) found that individuals who recovered from a SUD were as likely to be employed and work full-time as individuals with no history of a SUD, they also found higher rates of involuntary job loss among those who recovered from a SUD than among those with no SUD history. This pattern of findings might result from the visibility of the former SUD.

Goffman (1963) discussed a distinction between visible (race, physical disability) and invisible (SUDs, major depression) stigmas. Individuals with a visible stigma are discredited because their stigma is readily discernible by others. In contrast, those with an invisible stigma are discreditable but not discredited until their stigma becomes disclosed to others. The Baldwin et al., (2010) findings are consistent with former SUDs representing an invisible stigma during the hiring process. It is unlikely that a job applicant will disclose a former SUD in this situation to avoid discrimination in hiring. Therefore, obtaining employment may not be affected by a former SUD. Once employed, however, a former SUD may become either involuntarily or voluntarily disclosed. Any stigmatization that follows may adversely affect the individual’s employment experience and productivity outcomes (lower performance and more absenteeism), eventually leading to involuntary termination. Unfortunately, Baldwin et al., (2010) did not have information on the former SUD’s disclosure status.

However, a national study of individuals who recovered from a SUD addressed the disclosure issue. Vilsaint et al., (2020) asked participants if they had experienced various types of micro- and macro-discrimination “because someone knew about your alcohol or drug use history” (p. 3). The most common micro-discriminations were others expecting the person to relapse (49%), social exclusion (being ignored—30%; being avoided—36%), adverse interpersonal treatment (treated less favorably—37%, disrespected—34%, treated like a criminal—29%, treated as dangerous—25%), and being held to a higher standard (38%). Three of the most common macro-discriminations directly related to employment were an inability to find a job (16%), losing a job (15%), and failure to obtain a promotion (12%).

Despite research highlighting the deleterious effects of stigma at work, we know little about the impact of workplace stigma on SUD treatment and recovery outcomes. However, the 2018 National Survey on Drug Use and Health revealed that among individuals in need of SUD treatment, 37.8% did not seek treatment because of stigmatization-related concerns—17.2% because others would have a negative opinion of them and 20.6% because it might harm or jeopardize their job (Substance Abuse and Mental Health Services Administration, 2019). In addition, a small body of research on public exposure to stigmatizing attitudes and behaviors suggests it creates a critical barrier to recovery. By undermining recovery capital and increasing motivation to avoid pejorative labels and discrimination, stigmatization resulting from SUDs may (a) decrease initiation of unassisted and treatment-assisted recovery efforts; (b) increase the likelihood of failed self-change efforts, treatment attrition, and poor treatment outcomes; and (c) reduce the sustainability of recovery efforts (Buchanan & Young, 2000; da Silveira et al., 2018; Luoma et al., 2014; Marlatt & Witkiewitz, 2005; National Academies of Sciences-Engineering-Medicine, 2016a; Worley, 2017). These results suggest that SUD stigma in the workplace would undermine the likelihood that individuals approach their employers or union officials for help or take advantage of existing employer/union-sponsored programs (e.g., employee assistance programs, member assistance programs, employee resource groups). However, we are not aware of any research directly addressing this issue.

##### Research Proposition 4

Exposure to SUD stigmatization at work will decrease the likelihood of (a) initiating unassisted and treatment-assisted recovery efforts, (b) achieving or sustaining abstinence, and (c) achieving broad recovery outcomes such as growth in positive recovery capital and improved physical, psychological, and social well-being.

### Workplace Recovery Supports

#### Positive work conditions.

Positive work conditions represent events and experiences that are pleasant and may act as work-related resources. These resources allow individuals to “conduct and complete their work, meet expected job demands, accomplish goals, value their work, and ultimately experience higher levels of engagement” (Lee et al., 2020, p. 27). Positive work conditions include fair treatment, recognition, meaningful work, skill variety, autonomy, acquisition and use of skills, fair rewards, promotion opportunities, friendship formation, and social support from coworkers and supervisors.

Although research is limited, positive work conditions may support SUD recovery in two ways. First, by removing the motivation to self-medicate, they may reduce substance misuse, increase abstention, and reduce relapse (Albertsen et al., 2006; Frone, 2015). Therefore, positive work conditions may minimize the development or perpetuation of SUDs and promote recovery by reducing the likelihood of returning to substance use and increasing recovery’s sustainability by reducing relapse. The second way positive work conditions may affect SUD recovery is by increasing positive emotions and developing resources essential to recovery and well-being. Affective events theory (Weiss & Cropanzano, 1996) proposes that positive work events and experiences lead to positive emotions. Further, the broaden-and-build theory (Fredrickson, 2004) asserts that positive emotions *broaden* an individual’s thought-action repertoire involving awareness, creativity, thought processes, and action tendencies. This broadening of cognition and action allows individuals to *build* skills and resources that represent enduring reserves used in the future to build resilience and optimal functioning and increase physical, mental, and social well-being. Consistent with affective events and broaden-and-build theories, research supports associations of positive work conditions to higher positive affect, physical health, mental health, job satisfaction, and work engagement (Balducci et al., 2011; Chiaburu & Harrison, 2008; Frone & Blais, 2020; Vantilborgh et al., 2016; Viswesvaran et al., 1999).

##### Research Proposition 5

Positive work conditions will increase the likelihood of (a) initiating unassisted and treatment-assisted recovery efforts, (b) achieving or sustaining abstinence, and (c) achieving broad recovery outcomes such as growth in positive recovery capital and improved physical, psychological, and social well-being.

#### Workplace social control.

Social control theory posits that strong bonds with conventional social institutions (e.g., work organization) motivate individuals to engage in responsible behavior and refrain from substance misuse. In contrast, weak social bonds lead to substance misuse and other non-normative behavior. Strong social bonds result from monitoring and shaping normative behaviors by groups and institutions (Moos, 2007a, b). Likewise, Roman (1980, p. 407) stated that workplace social control is “exercised through socialization, patterns of reward distribution, and efforts to identify and control deviant behavior.” Therefore, the workplace social control framework posits that substance misuse is reduced when employees are regulated by and integrated into the work organization (Ames & Moore, 2016; Frone, 2013, 2019).

Two general types of workplace social control exist: formal and informal (Frone, 2013, 2019). Formal social control represents workplace policies and their enforcement regarding the use of substances and impairment during the workday and handling prescription drugs in some occupations. Prior research provides inconsistent evidence that a workplace policy on substance use will have a deterrent effect by itself (Ames & Moore, 2016; Frone, 2013; Pidd et al., 2016). However, a formal workplace substance use policy combined with enforcement may have a deterrent effect (Ames & Moore, 2016; Frone, 2013, 2019). A national study of U.S. workers found that when employees believed their supervisor could identify and was willing to address workplace substance use problems, they reported lower levels of alcohol and illicit drug use before and during the workday, as well as lower levels of illicit drug use off the job (Frone & Trinidad, 2012). Finally, a study found that required witnessing of the disposal of prescription drugs and written documentation of their administration were both negatively associated with prescription drug misuse among registered nurses (Trinkoff et al., 1999).

Informal social control represents the structural and contextual features of work that increase job performance visibility, thereby increasing the likelihood of detection by others if one is using or impaired by alcohol or other drugs at work (Trice & Sonnenstuhl, 1988). Job visibility increases with low mobility during work hours, high task interdependence, and direct contact with coworkers or organizational outsiders (e.g., customers, clients, patients, and the general public). The limited direct research on informal social control shows that performing one’s job in public, working closely with others, and low job mobility are associated with reduced substance misuse during the workday and away from work (Frone, 2003; Macdonald, Wells, & Wild, 1999).

Finally, a work-related mechanism exists within unionized workplaces that has the potential to leverage informal social control to help individuals with SUDs develop the motivation to begin the process of treatment and recovery. This mechanism represents member assistance programs (MAPs). MAPs are voluntary, peer-based programs that help motivate union members to seek help for SUDs and other personal problems, as well as provide support and assistance to those seeking help and those in recovery (Bacharach et al., 1996, p. 261). Peer-based counselors are union members who voluntarily assist coworkers in solving personal problems during their regular workday. They may confront coworkers to help them accept that they have a problem and need help, provide referrals for treatment, and often informally monitor a person’s progress through treatment, and offer post-treatment support to help ensure a successful recovery (Bacharach et al., 2001).

Although union members with SUDs may directly approach peer counselors for help, most will not initiate this interaction due to high levels of denial associated with SUDs (Bacharach et al., 2001). Therefore, in terms of informal social control, peer counselors may approach an individual after receiving information from coworkers about performance problems. For example, the peer counselor may approach the individual and relay that their coworkers were concerned about their well-being after noticing some performance issues. The peer counselor can offer information and treatment referrals for any problem affecting the individual’s performance. Being approached this way makes the individual aware that their substance use may be noticeable to coworkers. Although peer counselors do not have disciplinary power, armed with reliable evidence of chronic performance problems, they may exert informal leverage over an individual by expressing concern for the individual’s well-being, describing the impact of their behavior on coworkers, and informing the individual that if they do not work with the MAP to address the source of their performance problems, the union might not be able to save the individual’s job if management gets involved (Bacharach et al., 2001).

Although there is a paucity of systematic, quantitative research on the role of workplace social control mechanisms in the SUD recovery process, the above research suggests that workplace social control may play a supportive role in motivating employees with substance use problems to seek treatment and helping those in recovery to maintain abstinence. Formal and informal workplace social control also may increase bonding with the work organization (i.e., increased commitment to coworkers and the organization) and levels of task and contextual performance, thereby supporting the development of human, social, and personal capital (see Table 3) that is critical to the initiation and sustainability of SUD recovery.

##### Research Proposition 6a

Formal workplace social control targeting substance misuse will increase the likelihood of (a) initiating unassisted and treatment-assisted recovery efforts, (b) of achieving or sustaining abstinence, and (c) achieving broad recovery outcomes such as growth in positive recovery capital and improved physical, psychological, and social well-being.

##### Research Proposition 6b

Informal workplace social control—structural and contextual features of work that increase the visibility of work performance—will increase the likelihood of (a) initiating unassisted and treatment-assisted recovery efforts, (b) achieving or sustaining abstinence, and (c) achieving broad recovery outcomes such as growth in positive recovery capital and improved physical, psychological, and social well-being.

#### Organizational Support.

Organizations can embody either a marginal capital or a human capital view of employees. The marginal capital view characterizes employees as low value, disposable, and lacking talent and drive. In contrast, the human capital view characterizes employees as making valuable contributions, warranting time and resources to develop skills, and only discharged under exceptional circumstances (Eisenberger & Stinglhamber, 2011). Organizational support theory posits that employees develop a general perception of how an organization values their contributions and cares about their well-being, referred to as perceived organizational support (POS; Eisenberger & Stinglhamber 2011). Employees who report high levels of POS also report work environments consistent with the human capital view, where there are high levels of positive work conditions and low levels of negative work conditions (Kurtessis et al., 2017).

Perceived organizational support creates a social bond between employees and organizations based on social exchange. Individuals reciprocate the organization’s valuing of their contributions and well-being by increasing performance and their affective attachment to the organization (Kurtessis et al., 2017). Social bonding with the organization also increases because high POS fulfills socio-emotional needs, such as approval, esteem, affiliation, and emotional support (Eisenberger & Stinglhamber, 2011; Kurtessis et al., 2017). As with the social control framework discussed earlier, a strong social bond with the organization may motivate individuals to engage in responsible behavior and refrain from substance misuse that can undermine their job performance. The difference between social control theory and organizational support theory is the process underlying the social bond. In the social control framework, a social bond with the organization develops through organizational monitoring and shaping normative behavior. In contrast, in the organization support framework, employees develop a social bond with the organization because it values employees’ contributions and well-being and provides a work environment that allows employees to flourish (Eisenberger & Stinglhamber, 2011; Kurtessis et al., 2017).

Consistent with expectations based on organizational support theory, one study reported a negative association between POS and employee alcohol misuse among military personnel (Wright et al., 2012). A second study reported that high POS indirectly reduced alcohol use among retail store employees by reducing levels of anger (O’Neill et al., 2009). Although no research has explored the association of POS with SUD recovery, there are reasons to believe that POS may support recovery. Organizations with high POS have work environments that promote positive work conditions and minimize negative work conditions. As discussed earlier, such work environments may increase the likelihood that those with current SUDs will initiate unassisted or treatment-assisted recovery efforts and reduce the likelihood that those in recovery will return to substance use. Moreover, relative to low POS organizations, high POS organizations are more likely to build the human, physical, and social capital critical to the initiation and sustainability of SUD recovery and individual well-being (Eisenberger & Stinglhamber, 2011; Kurtessis et al., 2017).

##### Research Proposition 7

High perceived organizational support will increase the likelihood of (a) initiating unassisted and treatment-assisted recovery efforts, (b) achieving or sustaining abstinence, and (c) achieving broad recovery outcomes such as growth in positive recovery capital and improved physical, psychological, and social well-being.

## Conclusion

SUDs represent critical public health and occupational health issues. Evidence suggests that many individuals eventually recover from their SUD and develop productive and meaningful lives. However, the path to recovery is a complex, dynamic, and heterogeneous process that unfolds over time in environmental contexts that can undermine or facilitate SUD recovery initiation and sustainability. An environmental context that broadly affects most adults’ lives is employment. Consistent with this impact, employment represents a critical resource in SUD recovery (e.g., Borrelli et al., 2017; Brown & Ashford, 2019; Grover & Paylor, 2010; Laudet & White, 2010; Monaghan & Wincup, 2013; Room, 1998; Worley, 2017). However, discussions of employment as a SUD recovery resource have not considered that employment also may undermine recovery.

This article’s overall goal was to articulate the potential impact of the employment context in SUDs. To address this goal and inform occupational health researchers and related professionals, we (a) described the nature of a SUD, (b) summarized definitions of SUD recovery and broad themes associated with recovery, (c) developed a working definition of workplace supported recovery, (d) presented a general heuristic model highlighting where work conditions may affect the SUD recovery process, and (e) developed research propositions to guide future research on how two sets of work conditions—workplace recovery hazards and supports—may undermine or facilitate SUD recovery. However, our heuristic model and associated research propositions represent an initial attempt to identify potentially critical directions for occupational health research on workplace supported SUD recovery.

Future research testing and extending the research proposed in this article may provide a better understanding of the positive and negative impact of the work environment on employees’ ability to recover from SUDs. Using a broad set of recovery outcomes, including workplace productivity outcomes, would enhance the bottomline case that employers and employees could benefit if employers directly address SUD recovery. Although the research propositions highlight the *general associations* of workplace recovery hazards and supports to SUD recovery outcomes, future research needs to uncover which workplace hazards and supports are most central to workplace supported recovery. Moreover, research needs to explore how the various work conditions operate together to undermine or support SUD recovery. A lack of research precluded a detailed discussion of two processes central to this issue—moderation and mediation (Frone, 1999, 2019). For example, regarding moderation, high levels of POS may decrease (buffer) the negative impact of workplace SUD stigma on SUD recovery outcomes. Relatedly, research on the processes linking the workplace to SUD recovery can examine heterogeneity across the various special emphasis populations described earlier (see Recovery Theme 3). In terms of mediation, negative work conditions and workplace physical availability of substances may increase workplace norms and rituals supportive of substance use, which reduces the likelihood of recovery among those with a current SUD and increases the likelihood of a SUD recurrence among those in recovery. Finally, future research on workplace supported recovery should endeavor to use longitudinal observational designs and randomized clinical trials to provide more robust causal evidence to motivate employers to consider policies and interventions to address SUD recovery among their employees.

This review demonstrates that little conceptual or empirical work exists on the intersection of work and SUD recovery. In other words, this issue represents an underdeveloped research area where occupational health researchers can have a critical impact on a significant societal and occupational health issue. The proposed research agenda can help occupational health researchers and practitioners develop, refine, and evaluate interventions that leverage the workplace to support and sustain SUD recovery.

## Figures and Tables

**Table 1:** Employment Rate Among Adults with a Substance Use Disorder and the Prevalence of Substance Use Disorders, Receiving Substance Use Treatment, and Recovery from a Substance Use Problem in the U.S. Workforce (2018, Estimated Population Total = 155,904,219)

Substance	Employment Rate Among U.S. Adults with a Substance Use Disorder^a^ / Population Total (Past Year)			Prevalence of a Substance Use Disorder in the U.S. Workforce^a^ / Population Total (Past Year)				Prevalence of Receiving Substance Use Treatment in the U.S. Workforce^c^ / Population Total (Past Year)				Prevalence of Being in Recovery or Recovered from a Past or Present Alcohol or Drug Use Problem in the U.S. Workforce^d^ / Population Total			
	Age Group			Age Group				Age Group				Age Group			
	18+	18–25	26+	18+		18–25	26+	18+		18–25	26+	18+		18–25	26+
**Alcohol or Illicit Drugs** ^**b**^	70.35%	71.82%	69.82%	8.71%		15.87%	7.45%	1.20%		1.52%	1.14%	8.51%		5.13%	9.10%
	13,575,861	3,687,065	9,888,796	13,575,861		3,687,065	9,888,796	1,871,822		354,127	1,517,695	13,263,560		1,192,736	12,070,824
**Alcohol**	74.32%	75.85%	73.84%	6.81%		11.11%	6.06%	0.85%		0.96%	0.83%	NA		NA	NA
	10,623,406	2,579,796	8,043,610	10,623,406		2,579,796	8,043,610	1,321,561		223,709	1,097,852				
**Illicit Drugs** ^**b**^	61.81%	66.93%	59.05%	2.96%		7.52%	2.16%	0.65%		0.80%	0.62%	NA		NA	NA
	4,611,634	1,747,895	2,863,739	4,611,634		1,747,895	2,863,739	1,011,044		184,871	826,173				

NA = Not Assessed

Estimates for employment rates, substance use disorders, treatment, and recovery are from the 2018 National Survey of Drug Use and Health, calculated using the Substance Abuse and Mental Health Services Administration (SAMHSA) public online data analysis system (PDAS). https://pdas.samhsa.gov/#/

a Substance use disorder is defined as meeting criteria for illicit drug or alcohol dependence or abuse based on definitions in the fourth edition of the *Diagnostic and Statistical Manual of Mental Disorders* (American Psychiatric Association, 2000).

b Illicit drugs refer to the use of cannabis, cocaine, hallucinogens, inhalants, methamphetamine, and heroin, and the nonmedical use of prescription pain relievers, stimulants, tranquilizers, and sedatives.

c Substance use treatment for each class or combination of substance use classes refers to receiving treatment in any of the following locations in the past 12 months or receiving treatment for medical problems associated with substance use: (1) a hospital overnight stay as an inpatient, (2) a residential drug or alcohol rehabilitation facility where they stayed overnight, (3) a drug or alcohol rehabilitation facility as an outpatient, (4) a mental health center or facility as an outpatient, (5) an emergency room, (6) a private doctor’s office, (7) a prison or jail, (8) a self-help group (e.g., Alcoholics Anonymous or Narcotics Anonymous), or (9) some other place.

d Individuals were asked whether they thought they ever had a problem with their use of alcohol or other drugs. Individuals who thought they had a problem with their alcohol or other drug use were asked whether they considered themselves to be in recovery or to have recovered from the problem with their alcohol or other drug use.

**Table 2:** Definitions of Recovery

Source	Description
Betty Ford Institute Consensus Panel (2007, 2009)	Recovery from substance dependence is a voluntarily maintained lifestyle characterized by sobriety, personal health, and citizenship. *Sobriety* refers to abstinence from alcohol and all other nonprescribed drugs. *Personal health* refers to improved quality of personal life. *Citizenship* refers to not just a cessation of socially harmful behaviors, but also the development of pro-social behaviors (e.g., living a productive life, helping others).
Office of the Surgeon General (2016)	A process of change through which individuals improve their health and wellness, live a self-directed life, and strive to reach their full potential. Even individuals with severe and chronic substance use disorders can, with help, overcome their substance use disorder and regain health and social function. This is called remission. When those positive changes and values become part of a voluntarily adopted lifestyle, that is called “being in recovery.” Although abstinence from all substance misuse is a cardinal feature of a recovery lifestyle, it is not the only healthy, pro-social feature.
UK Drug Policy Commission Recovery Consensus Group (2008)	The process of recovery from problematic substance use is characterised by voluntarily-sustained control over substance use which maximises health and wellbeing and participation in the rights, roles and responsibilities of society.
Recovery Science Research Collaborative (Ashford et al., 2019)	Recovery is an individualized, intentional, dynamic, and relational pro- cess involving sustained efforts to improve wellness.
White (2007)	Recovery is the experience (a process and a sustained status) through which individuals, families, and communities impacted by severe alcohol and other drug (AOD) problems utilize internal and external resources to voluntarily resolve these problems, heal the wounds inflicted by AOD-related problems, actively manage their continued vulnerability to such problems, and develop a healthy, productive, and meaningful life.

**Table 3:** Dimensions of Recovery Capital (Cloud & Granfield, 2008; White & Cloud, 2008)

Dimension	Description
Human capital	Individuals’ internal assets, such as genetic inheritance, knowledge, skills, education, employability, self-esteem and self-efficacy, motivation to change, functional coping styles, spirituality, sense of meaning in life, as well as a lack of comorbid physical and mental health issues and higher physical and mental well-being.
Social capital	The sum of resources, reciprocal obligations, and benefits that accrue to an individual by virtue membership in social networks, such as social norms, availability of various types of social support, and access to opportunities through family, friends, coworkers, and employers.
Physical capital	Tangible economic or financial assets, such as income, savings and investments, stable housing, food security, transportation, and insurance, which provide financial stability and access to other important resources and life options.
Cultural capital	Cultural values, beliefs, norms, and perceptions that support conformity to dominant social behaviors.
Community capital	Attitudes, policies, and resources in a community related to SUDs and the promotion of recovery, such as efforts to reduce SUD and recovery-related stigma, visible and diverse recovery role models, acute and long-term systems of SUD treatment, accessible and diverse recovery mutual aid resources, recovery community support institutions (e.g., recovery centers, schools, and homes; recovery industries and sheltered workplaces; and recovery ministries and churches), and sources of sustained recovery support and early re-intervention (e.g., recovery checkups in treatment programs, employee assistance programs, professional assistance programs, drug courts.

**Table 4:** Prevalence of Employment-related Positive and Negative Experiences during Active Addiction and Recovery

	U.S.^a^ (N = 3,208)		Canada^b^ (N = 855)		UK^c^ (N = 802)		Australia^d^ (N = 573)	

**Positive Experiences**	**Active Addiction**	**Recovery**	**Active Addiction**	**Recovery**	**Active Addiction**	**Recovery**	**Active Addiction**	**Recovery**
Steadily employed	51%	83%	53%	79%	40%	74%	46%	73%
Positive job performance evaluations	49%	89%	41%	81%	33%	72%	32%	71%
Furthered education or training	37%	78%	34%	72%	32%	80%	33%	65%
Started own business	15%	28%	14%	26%	12%	18%	15%	26%
**Negative Experiences**								
Fired or suspended	51%	10%	44%	4%	50%	2%	38%	3%
Frequently missed work or school	61%	4%	61%	4%	56%	2%	56%	5%
Lost professional license	6%	1%	5%	1%	NA	NA	NA	NA
Dropped out of school or university	33%	4%	39%	3%	30%	4%	36%	4%

NA = Not asked

a Laudet (2013);

b McQuaid et al., (2017) ;

c Best et al., (2015);

d Best (2015)
